# Telemedicine in cardiovascular surgery during COVID‐19 pandemic: A systematic review and our experience

**DOI:** 10.1111/jocs.14933

**Published:** 2020-08-16

**Authors:** Ayomikun Ajibade, Hiba Younas, Mark Pullan, Amer Harky

**Affiliations:** ^1^ Birmingham Medical School University of Birmingham Birmingham UK; ^2^ St George's Medical School University of London London UK; ^3^ Department of Cardiothoracic Surgery Liverpool Heart and Chest Hospital Liverpool UK; ^4^ Department of Integrative Biology, Faculty of Life Sciences University of Liverpool Liverpool UK

**Keywords:** cardiac, coronavirus, COVID‐19, surgery, telemedicine, vascular

## Abstract

**Objective:**

The SAR‐COV‐2 pandemic has had an unprecedented effect on the UK's healthcare systems. To reduce spread of the virus, elective treatments and surgeries have been postponed or canceled. There has been a rise in the use of telemedicine (TM) as an alternative way to carry outpatient consultations. This systematic review aims to evaluate the extent to which TM may be able to support cardiac and vascular surgery patients in the COVID‐19 era.

**Methods:**

We looked into how TM can support the management of patients via triaging, preoperative, and postoperative care. Evaluations targeted the clinical effectiveness of common TM methods and the feasibility of applying those methods in the UK during this pandemic.

**Results:**

Several studies have published their evidence on the benefit of TM and its benefit during COVID‐19, the data related to cardiovascular surgery and how this will impact future practice of this speciality is emerging and yet larger studies with appropriate timing of outcomes to be published.

**Conclusion:**

Overall, the use of virtual consultations and remote monitoring is feasible and best placed to support these patients via triaging and postoperative monitoring. However, TM can be limited by the need of sophisticated technological requirement and patients’ educational and know‐how computer literacy level.

## 1 INTRODUCTION TO COVID‐19

In late 2019, a novel coronavirus, identified as SARS‐COV‐2 originated in the city of Wuhan, China.^1^, ^2^, ^3^ The coronavirus spread worldwide resulting in a global pandemic.^1^, ^2^ The disease caused by the novel coronavirus is commonly known as COVID‐19. On the 29th January, the first few cases of COVID‐19 were declared in England^4^ and since then there have been over 250 000 confirmed cases and over 40 000 COVID‐19 related deaths in the UK.^5^

COVID‐19 is a highly infectious disease with an original reproductive value of 2.5 to 3.5^6^, ^7^; infections can prompt cough, fever, and shortness of breath in symptomatic individuals.^1^ In more severe cases, COVID‐19 can result in acute respiratory distress syndrome or organ failure,^1^, ^2^ leading to some individuals being admitted to intensive care units. The high reproductive value of COVID‐19 combined with the limited treatment options or vaccine against the disease has led to an overrun of the UK's healthcare systems.^6^, ^7^

The COVID‐19 pandemic has influenced the conduct of society: as of 23 March 2020, the UK government announced a lockdown in which strict social distancing measures were to be followed by UK residents.^7^ New guidelines meant that residents could only leave for essential work, food shopping, and daily exercise. The lockdown also saw the shutdown of many entertainment and nonessential businesses such as restaurants, cinemas, and concert halls. Since then, as of 22nd May, the lockdown rules in the UK have been drastically relaxed and as the reproductive value fell under 1^8^ and lockdown measures across the globe have been less restrictive as the economy and society seeks to find a new “normal.”.

### 1.1 COVID‐19 and the impact on UK healthcare systems

The impact of the COVID‐19 pandemic on healthcare systems has been equally as disruptive.^7^ Critical care systems across western healthcare were originally overwhelmed by the outbreak. Over 250 000 volunteers and 5000 retired National Health Service (NHS) staff were recruited to help deal with the effects of the pandemic.^9^ There have been detrimental effects on other aspects of healthcare: regular cancer screenings were paused,^10^ and elective surgeries postponed in an attempt to contain the outbreak.^11^, ^12^ As of recently, guidelines have been released to start phasing these procedures back into practice,^13^, ^14^ however, it will be sometime before the impending backlog of surgeries is resolved.^10^

The cancellation of all except essential surgeries, together with guidelines to minimize contact between patients and physicians, had an impact on the practice of all major surgical specialities. Difficult decisions must be made where physicians must weigh up the risk of vulnerable patients being exposed to SARS‐COV‐2, vs the risk of delaying their surgical treatments. Because of this, there has been a call for the use alternative avenues, such as telemedicine (TM), that may be able to play a role in alleviating some of the impact of COVID‐19 by bridging the gap between patients and surgeons.^15^

### 1.2 Introduction to TM

TM refers to the use of information and telecommunication technologies to facilitate the remote provision of healthcare services. While the origin of TM can be traced back several hundred years, the modern form of TM was established in the 20th century, which saw the rapid evolution of increasingly sophisticated communication technology.^16^ A milestone in TM was reached when in the 1960s, the National Aeronautics Space Administration, used satellite communication in a spaceflight mission to enable doctors on earth to track the physiological changes of astronauts in space.^17^

The advent of the telephone, television, and two‐way audio and video communication channels played major roles in expanding the reach of TM. Now in more recent times, mobile technology and the internet have emerged and become mainstays, making TM remarkably more accessible in the modern era.^18^ The main categories of TM include *real‐time communication* (synchronous), for example, videoconferencing for online consultation and diagnosis; *store‐and‐forward communication* (asynchronous), for example, electronic transfer of clinical data such as images or medical history; and *remote monitoring*, which enables doctors to remotely observe and track a patient's real‐time clinical signs.^19^

The World Health Organization has highlighted the benefits of the use of rapidly developing information technologies in medicine, due to the potential scope of providing increasingly convenient, time‐saving, and perhaps more cost‐efficient doctor‐patient interaction.^20^ While it has been argued that TM cannot always be an adequate substitute for traditional face‐to‐face healthcare and physical examination,^21^ the advantage of the remote delivery of health services becomes clearly apparent in situations where the point of contention is the distance between the patient and physician. For example, TM has often been vital in linking rural and medically underserved areas to GPs and specialists.^17^, ^22^ Similarly, in the case of the current SARS‐Cov‐2 crisis, maintaining social distancing is vital to the suppression of the pandemic, and with the additional factor of the growing strain on NHS resources, these conditions have brought the value of TM to the forefront of medical practice.

### 1.3 TM use in the UK

Whilst TM has always had strong support on paper, the UK healthcare community at large has been slow in taking advantage of the resource. TM has had a limited history of use in the UK with regards to secondary and tertiary care, however, the current conditions have forced healthcare service providers across all frontiers to adapt to TM on an emergency basis.^23^ The NHS and the GMC have released resources and guidelines to assist both primary and secondary care clinicians in implementing online consultations where possible.^23^, ^24^, ^25^

TM has already been adapted into primary care, with telephone consultations and home‐monitoring systems already are in regular use in general practice, however, with the adjustments made to secondary and tertiary care services in the COVID‐19 era, it is unclear as to whether TM can meet the needs of complex specialities such as cardiovascular surgery. Thus, we will explore whether, in the COVID‐19 era, TM can meet the healthcare demands of cardiac and vascular surgery specialities.

### 1.4 Evaluating the usefulness of TM in cardiovascular surgery patients

For cardiac and vascular surgery, essential to the management of new and existing patients is effective triaging on the basis of the NHS guidelines for surgical prioritization during the pandemic.^26^ To practice healthcare within the social distancing guidelines, it is necessary to evaluate the potential of TM as a platform through which triaging and preoperative investigations can be remotely carried out in the context of cardiac surgery and vascular surgery.

A major aspect of the research surrounding the use of TM in cardiovascular settings has involved remotely‐based cardiac specialists providing expert diagnoses and assessment of patient condition from a distance, using patient history, live biodata, and cardiac imaging. Remote analysis of echocardiograms via real‐time teleconsultation was used to triage patients for cardiac surgery with good outcomes,^27^, ^28^ and the remote interpretation of results from a hand held ultrasound proved to be beneficial in the diagnosis of most cardiovascular diseases (CVDs) in comparison to standard echocardiography.^29^ Specialist teleconsultations of angiograms have also demonstrated accuracy in diagnosis and referral to procedures such as coronary artery bypass graft (CABG).^27^, ^30^ Similarly remote transmission of cardiac magnetic resonance imaging has been successfully used to assess congenital heart disease in children.^31^ However, the use of cardiac imaging in remote diagnosis requires the availability of imaging equipment and trained technicians to perform the scans before the images can be interpreted by a remote specialist. As the current crisis situation calls for minimum contact between patients and healthcare personnel, this category of TM technology for managing cardiac surgery candidates is of limited use amidst COVID‐19.

The electrocardiogram (ECG) is amongst the most pivotal tools used in the management of cardiac conditions, and has seen significant development over the years. Since 1906,^32^ substantial effort has gone into investigating the efficiency of a tele‐ECG interpretation, and most studies have found it to be just as effective, if not superior to a regular 12‐lead ECG, in the detection and triaging of patients with myocardial infarction and other complications.^33^, ^34^, ^35^ However, for the purpose of use during a pandemic like COVID‐19, tele‐ECG home‐monitoring technologies are more relevant. In more recent years, the contribution of mobile health (mhealth) technologies has transformed remote monitoring through the introduction of mobile phone applications that can transmit patient vitals straight to physicians, have patient‐friendly user interfaces, and are easily accessible. Single lead portable ECG devices such as the AliveCor Heart Monitor record ECG traces and transmit them to a mobile application with which they are paired, and can then be viewed by specialists.^36^ Such devices have been useful in detecting arrhythmias,^37^, ^38^ and thus have the potential to be used during COVID‐19 to remotely monitor heart rhythms of surgical candidates.

A significant amount of research has gone into investigating the role of portable and wireless home‐monitoring technologies such as AliveCor, in the remote monitoring of patients with cardiac conditions. One study used the ASCOLTA telemonitoring system to wirelessly collect biometric data from heart failure (HF) patients, including ECG, respiratory rate, and oxygen saturation (SpO_2_), along with questionnaires on general health data, and found that the combined information allowed cardiologists to effectively determine a patient's health status remotely and with high efficacy.^39^ Wireless wearable sensors are now increasingly available in the market, for measuring vital signs like heart rate (HR) and blood pressure (BP). One group developed a telemonitoring system connecting wearable sensors via Bluetooth to android mobile phones, which would send real‐time clinical data to a web interface accessed by doctors. Thus physicians were able to monitor the HR, BP, and body temperature of cardiac patients in real‐time, and after a comparison against data collected from traditional devices, the data from the wearable technology were deemed accurate. There was also an alarm system set up to warn of any changes or abnormalities detected.^40^ More recently another similar study also managed to record real‐time ECGs and SpO_2_ along with HR, BP, and temperature, via wearable circuits and developed a mobile phone application to receive and transmit the data to doctors.^41^

BP/HR monitors, pulse oximeters, scales, and ECG monitors are devices generally employed in the tele‐management of HF, which patients are taught to use at home, and the data from these are wirelessly transmitted to healthcare staff.^42^ Several HF trials such as the TIM‐HF2^43^ and BEAT‐HF^44^ have used these telemonitoring styles. Patient self‐monitoring has also lead to favorable outcomes in BP reduction compared to conventional follow‐ups,^45^ and one study also showed that physicians were able to make more frequent treatment adjustments in the self‐monitoring group, compared to the conventional group.^46^ Telemonitoring was also useful in reducing rehospitalization within 30 days of an acute MI.^47^ The DICE intervention was effectively carried out using remote biometrics monitoring to track the post‐CABG surgery health status of vulnerable patients, demonstrating that this could be implemented in place of conventional in‐person follow‐up appointments.^48^

While some trials show that superiority of using TM over conventional management may be debatable, the majority of the research indicates that the monitoring of clinical parameters of cardiac patients during COVID‐19 is likely to be useful in not only the overall management of the changing health status of patients, but also more specifically in facilitating effective remote presurgical triaging, detecting cardiac deterioration, and managing potential postsurgical complications. Some of the aforementioned HF trials also integrated structured telephone support in patient care, along with telemonitoring, to gather more detailed patient information and receive regular updates. Combined, these TM methods show evidence of reduced hospitalizations,^49^ and thus may have benefited during COVID‐19 in reducing patient exposure to hospitals which are SARS‐Cov‐2 hotspots, and in generally reducing doctor‐patient contact. Video consultations, combined with remote monitoring, were also a feasible replacement for standard ambulatory follow‐up after cardiac device implantation.^50^, ^51^ Moreover, telemonitoring of physiological and haemodynamic data from these implanted devices such as cardiac resynchronization defibrillators has also been extensively studied, as this data can indicate oncoming complications like cardiac decompensations. The IN‐TIME trial demonstrated the benefits of the use of such devices in HF patients with implants, in reducing all‐cause mortality and rehospitalizations.^52^ In a similar TELECART study, implant monitoring did not correlate with reduced all‐cause mortality, however, it was useful in making timely improvements to patient management and thus could potentially help reduce hospitalizations.^53^ Implanted devices that allow the monitoring of pulmonary artery pressure are also becoming more common, and once again, as demonstrated in the CHAMPION trial, enable physicians to track and make appropriate changes to treatment regimens in CVD patients, reducing hospitalizations and mortality.^54^

The feasibility of a home‐based cardiac rehabilitation program using TM, to replace the standard in‐hospital program for patients who have undergone cardiac surgery (eg, CABG, valvular surgeries), has also been tested. By providing patients with at‐home exercise equipment and a one‐lead tele‐ECG device, and through scheduled telephone and videoconferencing, hospital staffs were able to track patients for any complications, and facilitate effective physiotherapy.^55^, ^56^ While patients were still required to make hospital visits through the duration of the programs, these could possibly be minimized in a critical situation such as the current one. However, these programs would then be less suitable to patients with higher complication risks, who may require in‐hospital facilities and face‐to‐face support. The FIT@Home study conducted a 12‐week home‐based exercise training program for cardiac surgery patients, where patient status was tracked using a remote heart‐rate monitor and regular telephone support. The results were comparable to a conventional rehabilitation control group, and in‐person consultations were minimal throughout the study. However, only patients with low to moderate risk of adverse events were included, thus TM based rehabilitation may not be suitable for higher‐risk patients.^57^ Postoperative home rehabilitation may also be less than convenient to patients lacking at‐home caregivers.

With regards, to vascular surgery, TM has also proved practical and feasible. Surgical site infections are common among patients who have undergone vascular surgery, and thus wound monitoring is a significant aspect of post‐op care. Studies have utilized mobile and smartphone technology to assess surgical wounds, in place of standard outpatient care. The accuracy of physician analysis of wound images taken by smartphone and other digital cameras is comparable to that of in‐person wound examination.^58^, ^59^, ^60^ Mousa et al^59^ successfully used a home telemonitoring system (BP, HR, SpO_2_, etc) to also track patient status postarterial revascularization procedures. In another instance, videoconferencing was used for the management of patients at home after carotid endarterectomy procedures, which allowed for early patient discharge.^61^ Preoperative management and referral of vascular surgery candidates using TM has also been tested, involving live consultations with off‐site specialists, however, this usually requires patients to be physically present at locations which have access to trained staff and diagnostic facilities.^62^, ^63^, ^64^

### 1.5 TM cannot always be relied upon

TM is limited by the availability of technological resources. The rapid implementation of lockdown measures has led to an increased reliance on essential workers to deliver necessary electronic devices to workplaces and homes around the UK. The increased supply‐demand ratio has led to a lack of available resources which drive TM use, including laptops, the correct streaming bandwidth, and specialist communication software. With these resources needed for TM to even work, the implantation of this method of healthcare is likely to benefit the patients who already are in possession of useable and higher quality communication devices, as the rest of patients wait to catch up. Furthermore, TM in the COVID‐19 era may fail those who are not as technologically proficient or those who cannot financially support the use of high‐quality communication devices and software which benefit TM. Whilst this problem can be overcome using the help of free communication platforms such as Skype, WhatsApp, or Zoom, most of which are easily accessible to those with workable smartphones, there is a privacy concern surrounding these platforms which may put patients in harm's way.

The guidance given by the GMC recommends that TM should only be used in cases where the clinician is familiar with the patient and has a straightforward clinical need. Situations where the patient has more complex clinical needs or requires more delicate prescriptions such as injectables, then TM is no appropriate.^65^ Therefore, TM, whilst available, may not always be appropriate for use in healthcare especially when dealing with new patients who have more complex clinical needs. In the case of cardiac and vascular medicine, TM may be less appropriate in first‐time surgical referrals. However, under the difficult circumstances, physicians have been told that they may have to approach TM use with a “benefits outweigh the risk” policy; where there are situations of technology limitations or lack of resources, clinicians are permitted to use less secure communication platforms or alternative methods to bring healthcare into the homes of patients around the UK.^66^

A further limitation of TM is that preoperative evaluations are difficult to perform and a full physical examination cannot be conducted over a digital platform.^66^ Regardless of these issues, TM is seen to be just as effective as face‐to‐face consultations^67^ when it comes to patient satisfaction and clinical effectiveness regarding triaging, straightforward diagnosis, and postoperative rehabilitation. It is important to remember, however, that TM is always dependant on the availability and workability of the technology needed to power it.

Table 1 is a summary from key studies outlining the practice and use of TM and how this can affect clinical practice.

**Table 1:** Summary of key studies on telemedicine and its implications

No.	Ref	First author, year	Title	Summary of TM intervention	Summary of findings/conclusions	Potential for perioperative use in cardiovascular surgery
1	27	Gackowski,^27^ 2011	Development, Implementation, and Multicenter Clinical Validation of the TeleDICOM—Advanced, Interactive Teleconsultation System	Use of a tele‐DICOM system for remote diagnostic analysis of coronary angiograms and echocardiograms. Comparison with standard imaging.	Teleconsultations using the tele‐DICOM system allowed for accurate decision making for CABG in comparison to decisions made using standard imaging procedures.	Diagnosis, triage, and management in cardiac surgery patients.
2	28	Sekar,^28^ 2007	Telecardiology: Effective Means of Delivering Cardiac Care to Rural Children	Use of videoconferencing and tele‐echocardiography to carry out remote diagnoses, and decisions for surgery, in rural children.	Telecardiology was effective in facilitating off‐site decision making for cardiac surgery and allowed transmission of accurate images.	Diagnosis, triage, and management in cardiac surgery patients.
3	29	Evangelista,^29^ 2016	Hand‐held Cardiac Ultrasound Screening Performed by Family Doctors With Remote Expert Support Interpretation	Remote interpretation of a hand‐held cardiac ultrasound and comparison with standard echocardiography.	There was a good level of agreement between remote expert interpretation and standard echocardiography.	Diagnosis, triage, and management in cardiac surgery patients.
4	30	Bonvini,^30^ 2002	Telemedicine for cardiac surgery candidates	Tele‐transmission and remote interpretation of coronary angiograms to facilitate remote diagnosis and decision for surgical intervention. Comparison with direct scrutiny of angiograms.	Decisions based on remote transmissions were accurately made in 91% of the cases. There was a good level of diagnostic agreement between remote transmissions and direct scrutiny.	Diagnosis, triage, and management in cardiac surgery patients.
5	31	Garg,^31^ 2015	Remote Delivery of Congenital Cardiac Magnetic Resonance Imaging Services: A Unique Telemedicine Model	Tele‐transmission and videoconferencing to a facilitate the remote interpretation of Cardiac magnetic resonance imaging, in comparison to a conventional CMRI setting.	TM based CMRI services were efficacious and comparable to a conventional CMRI service.	Diagnosis, triage, and management in cardiac surgery patients.
6	33	Schwaab,^33^ 2006	Validation of 12‐lead tele‐electrocardiogram transmission in the real‐life scenario of acute coronary syndrome	Tele‐transmission and remote interpretation of a 12‐lead ECG, compared with ECGs taken via standard setting.	Tele‐ECGs allowed for accurate diagnosis of STEMI and were comparable to diagnoses made via a conventional ECG.	Diagnosis, triage, and management in cardiac surgery patients.
7	34	Mavrogeni,^34^ 2000	Supervision of thrombolysis of acute myocardial infarction using telemedicine	Tele‐transmission and remote interpretation of 12‐lead ECGs.	Tele‐ECGs were effective in the remote management of acute myocardial infarction.	Diagnosis, triage, and management of cardiac surgery patients.
8	35	Rasmussen,^35^ 2014	Diagnostic performance and system delay using telemedicine for prehospital diagnosis in triaging and treatment of STEMI	Use of tele‐ECG in prehospital cardiac triage.	Tele‐ECG was effective in aiding prehospital remote triage for cardiac surgery.	Diagnosis, triage, and management of cardiac surgery patients.
9	37	Chan,^37^ 2017	Head‐to‐head comparison of the AliveCor heart monitor and Microlife WatchBP Office AFIB for atrial fibrillation screening in a primary care setting	Use of single‐lead ECG smartphone monitor AliveCor in the remote detection of atrial fibrillation. Comparison with standard 12‐lead ECG.	Effective detection of AF in 66.7% of patients. Concerning a false‐negative rate of 33.3%, however, still has a high potential for supplementation in primary care.	Remote monitoring of heart rhythms in cardiac surgery candidates.
10	38	Garabelli,^38^ 2016	Comparison of QT Interval Readings in Normal Sinus Rhythm Between a Smartphone Heart Monitor and a 12‐Lead ECG for Healthy Volunteers and Inpatients Receiving Sotalol or Dofetilide	Use of single‐lead ECG smartphone monitor AliveCor in the remote monitoring of ECG trace. Comparison with standard 12‐lead ECG.	Effective remote monitoring detection of changes in the ECG signal was feasible and was comparable to results from a standard 12‐lead ECG.	Remote monitoring of heart rhythms in cardiac surgery candidates.
11	39	Romano,^39^ 2014	The informative contribution of the “virtual medical visit” in a new heart failure telemedicine integrated system	Use of wireless home‐monitoring technology and online questionnaires to track changes in health status of cardiac patients.	Remote monitoring system was effective in tracking the health status of HF patients.	Remote monitoring of changes in the vital signs and health status in cardiac surgery candidates.
12	40	Kakria,^40^ 2015	A Real‐Time Health Monitoring System for Remote Cardiac Patients Using Smartphone and Wearable Sensors	Use of Bluetooth‐enabled wearable sensor to remotely monitor cardiac parameters (HR, BP, body temperature).	Enabled effective monitoring and detection of changing cardiac vital signs. Accuracy of data from remote sensors was comparable to data from conventional devices.	Remote monitoring of changes in vital signs and health status in cardiac surgery candidates.
13	41	Al‐Naggar,^41^ 2019	Design of a Remote Real‐Time Monitoring System for Multiple Physiological Parameters Based on Smartphone	Use of wearable sensors and wireless smartphone technology to remotely track health status, including ECG readings, HR, etc.	Allowed accurate tracking of cardiac parameters, with low time delays in data transmission using Wi‐Fi.	Remote monitoring of cardiac parameters and health status in cardiac surgery candidates.
14	43	Koehler,^43^ 2018	Efficacy of telemedical interventional management in patients with heart failure (TIM‐HF2): a randomized, controlled, parallel‐group, unmasked trial	Use of home telemonitoring systems in heart failure patients to track ECG readings, BP, weight, and SpO_2_, combined with structured telephone support.	Remote management of heart failure patients via telemonitoring was feasible and led to improved patient care and patient outcomes.	Remote monitoring and management of cardiac parameters and health status in cardiac surgery candidates.
15	44	Ong,^44^ 2016	Effectiveness of remote patient monitoring after discharge of hospitalized patients with heart failure the better effectiveness after transition‐heart failure (BEAT‐HF) randomized clinical trial	Wireless home telemonitoring (BP, HR weight, etc.) and structured telephone support for management of heart failure patients.	Remote management using telemonitoring was as effective as standard care with no significant difference in primary outcomes.	Remote monitoring and management of cardiac parameters and health status in cardiac surgery candidates.
16	45	Bray,^45^ 2010	Does self‐monitoring reduce blood pressure? Meta‐analysis with meta‐regression of randomized controlled trials	Patient self‐monitoring of blood pressure.	Self‐monitoring of blood pressure is feasible and led to a reduction in blood pressure compared to usual care.	Remote monitoring of cardiac parameters and health status in cardiac surgery candidates.
17	46	McGillicuddy,^46^ 2013	Mobile health medication adherence and blood pressure control in renal transplant recipients: A proof‐of‐Concept randomized controlled trial	Wireless, remote blood pressure monitoring, self‐management system.	Telemonitoring of blood pressure led to improved quality of patient care and reduced blood pressures.	Remote monitoring and management of health status in cardiac surgery candidates.
18	47	Ben‐Assa,^47^ 2014	Is telemedicine an answer to reducing 30‐d readmission rates post‐acute myocardial infarction?	Use of home telemonitoring technology to manage cardiac patients.	Telemonitoring led to improved outcomes in MI patients.	Remote monitoring and management of vital signs in cardiac surgery candidates.
19	48	Kleinpell,^48^ 2015	Randomized Trial of a Discharge Planning and Telehealth Intervention for Patients Aged 65 and older after Coronary Artery Bypass Surgery	Remote home biometrics monitoring and telephone support for management of health status post‐CABG surgery (Discharge Intervention for Cardiac Elderly [DICE]).	Overall remote home monitoring was as effective as usual postoperative management, with improved patients outcomes in certain groups.	Remote monitoring and management of cardiac parameters and health status in cardiac surgery candidates.
20	49	Inglis,^49^ 2017	Structured telephone support or noninvasive telemonitoring for patients with heart failure	Structured telephone support and telemonitoring in heart failure patients.	TM interventions were effective and led to improved primary patient outcomes.	Remote monitoring and management of cardiac surgery candidates.
21	50	Sponga,^50^ 2018	Teleconsultation for left ventricular assist device patients: a new standard of care	Remote biometrics monitoring and videoconferencing for patient management postcardiac procedure	Remote interventions allowed effective tracking of patient status and improved quality of care	Remote postoperative management of patient health status.
22	51	Dalouk,^51^ 2017	Outcomes of Telemedicine Video‐Conferencing Clinic Versus In‐Person Clinic Follow‐Up for Implantable Cardioverter‐Defibrillator Recipients	Remote patient monitoring postcardiac device implantation via videoconferencing.	TM based postoperative management led to no significant differences in primary and secondary outcomes compared to usual care.	Remote postoperative management of patient health status.
23	52	Hindricks,^52^ 2014	Implant‐based multiparameter telemonitoring of patients with heart failure (IN‐TIME): A randomized controlled trial	Remote biometrics monitoring of data from implantable cardioverter‐defibrillators (ICDs) and cardiac resynchronization defibrillators (CRT‐Ds).	Telemonitoring was efficacious, feasible, and led to significant improvement in patient outcomes (lower mortality).	Remote monitoring to manage cardiac surgery patients with implanted devices.
24	53	Sardu,^53^ 2016	Telemonitoring in heart failure patients treated by cardiac resynchronization therapy with defibrillator (CRT‐D): the TELECART Study	Remote monitoring of health status of heart failure patients using data from ICDs.	Remote biometric data was a good predictor of hospitalization, and reduced HF‐related hospitalizations, however no reduction in mortality was seen compared to control group.	Remote monitoring in the management of cardiac surgery patients with implanted devices.
25	54	Givertz,^54^ 2017	Pulmonary Artery Pressure‐Guided Management of Patients With Heart Failure and Reduced Ejection Fraction	Telemonitoring of pulmonary artery pressures in HF patients with CardioMEMS implants.	Remote monitoring of implanted devices data led to a reduced rate of both hospitalization and mortality.	Remote monitoring to manage cardiac surgery patients with implanted devices.
26	55	Scalvini,^55^ 2009	Home‐based exercise rehabilitation with telemedicine following cardiac surgery	Remotely supervised, home‐based, postcardiac surgery rehabilitation program with telephone support and wireless ECG transmission.	Home‐based rehabilitation was effective and could be a feasible alternative to a standard in‐hospital program for certain patient groups.	Remote postoperative rehabilitation of cardiac surgery patients.
27	56	Scalvini,^56^ 2013	Home‐Based Versus In‐Hospital Cardiac Rehabilitation After Cardiac Surgery: A Nonrandomized Controlled Study	Remotely supervised, home‐based, postcardiac surgery rehabilitation program with telephone and video‐conference support, and wireless ECG transmission.	Home‐based rehabilitation via TM was feasible (for certain patient groups), with few differences between the TM group and control group, and results comparable to the standard in‐hospital program.	Remote postoperative rehabilitation of cardiac surgery patients.
28	57	Kraal,^57^ 2014	Effects of home‐based training with telemonitoring guidance in low to moderate risk patients entering cardiac rehabilitation: Short‐term results of the FIT@Home study	Home‐based, postcardiac surgery rehabilitation program with telephone support and wireless HR monitoring.	Home‐based rehabilitation via TM was effective and comparable to the center‐based control group program.	Remote postoperative rehabilitation of cardiac surgery patients.
29	58	Wiseman,^58^ 2016	Inter‐rater agreement and checklist validation for postoperative wound assessment using smartphone images in vascular surgery	Remote surgical wound management based on digital images taken on smartphone, compared with in‐person assessment.	Inter‐rater concordance was high between digital and in‐person groups for wound assessment and management decisions.	Remote postoperative wound monitoring for vascular surgery patients.
30	59	Mousa,^59^ 2019	Results of Telehealth Electronic Monitoring for Post Discharge Complications and Surgical Site Infections following Arterial Revascularization with Groin Incision	Home‐based telemonitoring systems to track wound status and general vital signs in patients post vascular surgery, in comparison to usual care.	TM based postoperative wound care was feasible with no overall differences in outcomes in TM vs control group. TM based wound care led to improved patient quality of life and increased satisfaction with care quality.	Remote postoperative wound management for vascular surgery patients.
31	60	Totty,^60^ 2018	Use of photograph‐based telemedicine in postoperative wound assessment to diagnose or exclude surgical site infection	Use of digital images for remote postoperative wound assessment. Compared with in‐person wound assessment.	High concordance between remote assessors and in‐person wound reviews.	Remote postoperative wound management for vascular surgery patients.
32	61	Robaldo,^61^ 2010	Telemedicine in vascular surgery: Clinical experience in a single center	Videoconferencing and remote BP monitoring for remote management of post endarterectomy patients.	Telemonitoring was feasible for remote postoperative management, with results comparable to the in‐hospital group, and allowed for early patient discharge.	Remote postoperative wound management for vascular surgery patients.
33	62	Hands,^62^ 2004	The use of telemedicine in the management of vascular surgical referrals	Digital images and videoconferencing for the diagnosis and triage of vascular surgery candidates.	TM allows for remote specialist assessment of vascular referrals with correct diagnoses.	Diagnosis, triage, and management in vascular surgery candidates.
34	63	Endean,^63^ 2001	Telemedicine in vascular surgery: Does it work?	Use of video‐surveillance and remote transmission of clinical parameters in the assessment of vascular surgery patients, compared with face‐to‐face assessment.	Accurate assessment and management by a remote specialist were feasible, with high inter‐rater reliability between the TM and in‐person assessors.	Diagnosis, triage, and management in vascular surgery candidates.
35	64	Lin,^64^ 2018	Implementation of a virtual vascular clinic with point‐of‐care ultrasound in an integrated healthcare system	Use of videoconferencing, remote imaging technologies in the diagnosis, management, and postoperative follow‐up of vascular surgery candidates.	Remote specialist management of vascular patients using TM is useful and effective, both pre and postoperatively	Diagnosis, triage, and management in vascular surgery candidates.

Abbreviations: BP, blood pressure; CABG, coronary artery bypass graft; ECG, electrocardiogram; HF, heart failure; HR, heart rate; TM, telemedicine.

### 1.6 Our experience

Since the lockdown has been introduced to United Kingdom on 23rd of March 2020, the healthcare provision in the entire NHS has been modified to accommodate the surge of COVID‐19 patients and minimize the risk of acquiring this disease. This has been achieved through isolation and maintaining social distances. This involved cancellation of all elective face‐to‐face clinics’ appointments and cardiovascular surgical interventions. One of the modalities that has been introduced to keep track of our patients and ensure their wellbeing are maintained is using TM and virtual follow‐ups. This has been the main approach, in particular, for outpatient clinics whether as new patient referral or routine follow‐up of those who have been discharged following their surgery.

At our unit, Liverpool Heart and Chest Hospital which is the regional hub for all cardiac surgery procedures in North‐West of England during COVID‐19 pandemic, we have used such practice successfully through virtual follow‐up using telephone conversation with patients who were due to come in for face‐to‐face review otherwise. We have also practiced the same method for patients who were due to be assessed for possible surgical intervention and if there was need for a face to face review, an arrangement would be made. Such practice has been welcomed by the clinical‐administrative staff while arranging for such telephone consultations. Patient experience has been positive so far and yet the clinicians have not reported any dissatisfaction from such practice.

It is important to reflect that such virtual clinic follow‐ups have its own advantage in lacking direct patient contact, therefore, no risk of COVID‐19 acquisition, especially vast majority of our cohorts are elderly patients who are at higher risk of acquiring COVID‐19. Nevertheless, virtual follow‐up has its own limitations in terms of lack of direct assessment of the clinical condition of the patient through routine examination and inspection of the surgical sites which could potentially lead to missing some manageable conditions as pleural effusion and unstable sternum. The long‐term impact of this practice is yet to be identified once this pandemic is over.

Additionally, our routine and ad‐hoc urgent multidisciplinary meetings (MDTs) have shifted to be virtual practice through using Microsoft Team application which has not only helped in maintaining social distance during this pandemic but also allowed more people to join the MDT. This practice not only applied for internal MDTs but also to the specialized ones that needed meetings of different specialists from the region to discuss complex cases which, before COVID‐19 pandemic, previously required travel of such specialists to a selected unit to meet and discuss such cases. Such initiation has provided convenience, maintained safe social distances, and saved travel times without affecting the decision making process and quality of services offered to patients.

## 2 CONCLUSION AND FUTURE RESEARCH

Overall, in the COVID‐19 era, remote monitoring shows the most potential for use in the ongoing crisis. Combinations of regular virtual consultations and remote monitoring of clinical parameters are feasible for cardiac surgery patients and would be useful to assess and triage before surgery. Remote monitoring could also be particularly useful in managing postoperative complications, to help reduce ambulatory visits and rehospitalizations for vascular surgery patients. This is especially vital right now as the social distancing measures are set to stay for the foreseeable future; the utmost priority is to explore the use TM to reduce patient to physician contact as much as possible.

As society and healthcare seek to find a “new normal,” greater investments will need to be made in technological gaps around the UK so that the use of TM can become more common. The authors believe that more attention is needed to see how TM can be made more accessible to those best placed to benefit from it.^68^, ^69^, ^70^, ^71^, ^72^, ^73^, ^74^, ^75^, ^76^, ^77^, ^78^, ^79^, ^80^, ^81^

In addition to this, a potential future for TM may be the use of telepresence surgery on vascular patients. Telepresence surgery would allow for more socially distanced operations to occur via TM and could benefit patients who cannot risk traveling longer journeys for operations. Whilst preliminary research has shown this type of surgery to be effective on swine,^68^ and robotic surgery is more widely accepted in healthcare, more research would be needed to see distanced operations could be applicable to humans and how feasible this surgery could be implemented under the NHS.

## CONFLICT OF INTERESTS

The authors declare that there are no conflict of interests.
